# Evaluation of the immune effect of foot-and-mouth disease virus-like particles derived from *Pichia Pastoris* on mice and pigs

**DOI:** 10.3389/fmicb.2025.1551395

**Published:** 2025-04-14

**Authors:** Zhiyao Li, Manyuan Bai, Shuanghui Yin, Yan Yang, Hu Dong, Zhidong Teng, Shiqi Sun, Endong Bao, Huichen Guo

**Affiliations:** ^1^College of Veterinary Medicine, Nanjing Agricultural University, Nanjing, China; ^2^State Key Laboratory for Animal Disease Control and Prevention, College of Veterinary Medicine, Lanzhou University, Lanzhou Veterinary Research Institute, Chinese Academy of Agricultural Sciences, Lanzhou, China; ^3^Agriculture and Rural Bureau of Yugur Autonomous County of Sunan, Zhangye, China; ^4^Gansu Province Research Center for Basic Disciplines of Pathogen Biology, Lanzhou, China

**Keywords:** foot-and-mouth disease, nascent-polypeptide-associated complex (NAC), virus-like particles, vaccines, *Pichia Pastoris*

## Abstract

Foot-and-mouth disease (FMD) is an acute, highly contagious, infectious disease that affects cloven-hoofed animals and the healthy development of animal husbandry. Despite the pivotal role of the inactivated FMD vaccine in preventing and controlling the disease, the production and preparation of the inactivated vaccine still present certain safety concerns. Virus-like particles (VLPs), which have a shell structure similar to that of the viruses but lack the genetic material of viruses, have emerged as a prominent area of research on developing next-generation FMD vaccines. In this study, co-expression of P1 and 3C was implemented to obtain the capsid protein of FMD virus (FMDV), and VLPs of FMD were prepared using *Pichia Pastoris*. Given that the enzymatic activity of 3C is not ideal in acidic yeast cells, the HLH pattern structure was added to the N-terminal end of 3C, which can be anchored near the exit of the nascent peptide chain of ribosomes. Furthermore, the alcohol oxidase (AOX) promoter, which regulates the expression of 3C, was enhanced by mutation. Then, FMDV VLPs were successfully produced in yeast. Immunization of mice and pigs with VLPs resulted in high levels of specific and neutralizing antibodies and provided protection against FMDV in pigs. In conclusion, FMDV VLPs can be successfully produced in *P. Pastoris*. This offers a new way to develop FMDV VLP vaccines.

## 1 Introduction

Foot-and-mouth disease (FMD) is a highly virulent infectious disease that considerably affects local animal husbandry. FMD can reduce animal production capacity and impose severe restrictions on local animal trade. FMD is still endemic in Asia, parts of Europe, Africa and South America, with developing countries bearing the brunt of the effect (Jamal and Belsham, 2013). FMD virus (FMDV) is categorized into seven serotypes, namely, A, O, C, Asia1, STA1, STA2, and STA3 and further subdivided into several subtypes (Jamal and Belsham, 2018). No cross-protection exists between serotype (Cao et al., 2014). Serotype C is becoming increasingly rare (Sangula et al., 2011), whereas serotypes A, O, and Asia1 remain widely prevalent (Rweyemamu et al., 2008). Serotypes STA1, STA2, and STA3 are typically prevalent in Africa (Knowles et al., 2016). FMDV can infect approximately 70 species of cloven-hoofed animals, including cattle, sheep, camels, and pigs (Quan et al., 2009; Osmani et al., 2019; Zhang et al., 2019). It spreads rapidly through contact, aerosols, and food (Paton et al., 2018). Although, the incidence of FMDV is 100% in susceptible animals, it rarely causes mortality (Diaz-San Segundo et al., 2017). Infected animals may have blisters on the mouth, nose, hooves, or udder of females or scabs that form after blistering (Wu et al., 2003). Efficacious vaccines, particularly safe and effective genetically engineered vaccines, are a crucial strategy to control FMD. These vaccines are the focus of current research.

Virus-like particles (VLPs) have a morphological structure similar to that of virions but lack viral genome: thus, they are an ideal vaccine form (Chackerian, 2007; Mignaqui et al., 2019). FMDV VLPs have been successfully produced in mammalian cells (Polacek et al., 2013), insect cells (Vivek Srinivas et al., 2016), *Escherichia coli* (Xiao et al., 2016), *Saccharomyces cerevisiae* (Le et al., 2024) and plant cells (Veerapen et al., 2018), but reports on its production in *Pichia Pastoris* are rare. Compared to mammalian cell expression systems, *P. pastoris* has a lower cultivation cost, requiring neither complex culture media nor strict cultivation conditions. Also, it grows rapidly, enabling the acquisition of a large quantity of expressed products within a short period. Regarding contamination prevention, the cultivation environment of *P. pastoris* is relatively simple and easy to control, leading to a lower contamination risk. In contrast, the cultivation of mammalian cells is vulnerable to contamination by bacteria or mycoplasma, etc., (Hervas-Stubbs et al., 2007). Compared with baculovirus expression systems, *P. pastoris* has relatively straightforward transformation and screening methods. It has lower experimental operation complexity and higher transformation efficiency. As a eukaryotic expression system, *P. pastoris* has an advantage over the *E. coli* expression system. It can fold and assemble proteins, which is more conducive to the correct assembly of VLPs. In contrast, proteins expressed by *E. coli* often form inclusion bodies and need a complex renaturation process. Additionally, proteins expressed by *P. pastoris* generally do not contain endotoxins, avoiding the impact of endotoxins on vaccine safety (Mamat et al., 2015). Nowadays, FMD is highly prevalent in developing countries. Yeast has the potential to be a valuable tool because of its low cost and modifiability. Numerous studies have been conducted on the preparation VLPs using *P. Pastoris*, such as enterovirus (Zhang et al., 2015), coxsackie virus (Feng et al., 2016), poliovirus (Xu et al., 2019; Sherry et al., 2020), and others. The assembly efficiency of VLPs has been a research interest for some time. Optimal expression systems and assembly methods are essential for producing VLPs.

Foot-and-mouth disease virus is a member of the *Picornaviridae* family and *Aphthovirus* genus. It lacks envelope, and has a genome of single-, positive-stranded RNA with a total length of 8,500 nts encoding the structural protein P1 and the non-structural proteins P2 and P3 (Ryan et al., 1989). Polyprotein P1 is cleaved by the 3C protease to VP0, VP3, and VP1. These then assemble into the icosahedral viral capsid (Robertson et al., 1985; Ryan et al., 1989; Jackson et al., 2002). Based on these findings, we investigated the potential of expressing FMDV VLPs in *P. Pastoris* by co-expressing P1 and 3C. The 3C protease causes host cell death (Belsham et al., 2000). Notably, this study revealed that 3C protease activity responds moderately to acidic conditions and has a minimal impact on yeast cell proliferation. In eukaryotes, nascent- polypeptide-associated complex (NAC), a molecular chaperone protein, comprises two subunits (α and β). The β NAC subunit is responsible for binding to ribosomes (Beatrix et al., 2000). The EGD1 gene encodes the β NAC subunit, and the 55 amino acids at its N-terminal end have an HLH structure with an α-helix flanked by a loop in the middle. Research has demonstrated that this structure can bind soluble proteins to yeast ribosomes both *in vivo* and *in vitro* (Wegrzyn et al., 2006). Subsequently, we regulated the processing of the P1 protein via the 3C by adding the HLH to the N terminus of the 3C. Additionally, we regulated 3C expression levels by strengthening alcohol oxidase (AOX) promoter that controls 3C (Hartner et al., 2008). Finally, we evaluated the immunogenicity of VLPs in mice and pigs.

## 2 Materials and methods

### 2.1 Strains, plasmids and reagents

The pPink-P1 (VP1 N17D + VP2 H145Y) acid-resistant mutant plasmid (Vázquez-Calvo et al., 2014) and pPink-3C (Supplementary material) plasmid was persevered by the Lanzhou Veterinary Research Institute, Chinese Academy of Agricultural Sciences. The PichiaPink Strain 1 (Invitrogen, Grand Island, NY, United States) was used expression P1 and 3C. The anti-FMDV polyclonal antibodies were persevered in our lab, while HRP-conjugated rabbit anti-pig IgG antibodies were purchased from Solarbio (Beijing, China).

### 2.2 Plasmids construction and strains screening

The HLH gene (sequence number X78725) was obtained from NCBI and, following sequence optimization was incorporated into the pPink-3C plasmid to create the pPink-H3C plasmid. The 712-777 region upstream of the AOX promoter of 3C was deleted with the primers D1-F/R, and the 190-203 region upstream of the AOX promoter of 3C was amplified with the primers A1-F/R (Table 1). Finally, the pPink-opH3C plasmid was constructed. The 3C cassette was digested from the pPink-3C plasmid by *Bgl*II/*Bam*HI and subsequently inserted into the pPink-P1 plasmid to construct pPink-P1/3C. Similarly, pPink-P1/opH3C plasmid was constructed. For transformation, plasmids were linearized by *Spe*I and then electrotransferred into the strain1. The transformation and screening according to the manufacturer’s instruction.

**TABLE 1:** Primer sequences used to modify the AOX promoter.

Primer	Sequence	Region
D1-F	TCCTCAACACCCACTTTAGGCTACTAACACCATGACTTTATTAGCCTGTC	–712 to –777
D1-R	GTGTTAGTAGCCTAAAGTGGGTGTTGAGGAGAAGAGGAGT	–712 to –777
A1-F	GCTGATAGCCTAACGTTCATGATCAAAATTTCATGATCAAAATTTAACTGTTC	–190 to –203
A1-R	GTTAAATTTTGATCATGAAATTTTGATCATGAACGTTAGGCTATCAGCAGTATTC	–190 to –203

### 2.3 SDS-PAGE and western blot detection

After induction, the strain was subjected to ultrasonic disruption at 350 W for 3 s with 3 s intervals for 20 min. The supernatant was then centrifugated at 12,000 rpm for 30 min. After electrophoresis, the gel was transferred to NC membranes for Western blot (WB) and incubated with 5% skim milk at room temperature for 2 h, washed three times with PBST, and then incubated overnight at 4°C with polyclonal antisera anti-FMDV and following this, incubated with HRP-conjugated anti-IgG for 1 h and then exposed for color development.

### 2.4 Purification and electron microscopic observation of VLPs

The supernatant obtained following the crushing and centrifugation was placed in assembly buffer for overnight dialysis (300 mM NaCl, 10 mM tris, 50 mM KCl, 2 mM MgCl, 1% Triton X-100, and 0.1 mM PMSF, pH 8.0). After centrifugation at 12,000 rpm for 30 min, 1 mL sample was added to the top of the 15%–50% (w/v) sucrose gradient solution and centrifuged at 35,000 rpm for 3 h. Then, the samples were separated and collected in 500 μL per tube from the top to the bottom. The absorbance was then determined using a UV spectrophotometer, and the samples with the highest absorbance were selected for transmission electron microscopic (TEM) observation and DLS analysis. Take 10 μL purified sample and add it onto 200-mesh copper grids, adsorbed for 2 min at room temperature, then washed with ddH2O, then stained with 3% phosphotungstic acid for 1 min. Finally, the samples were observed with a Hitachi H-7100FA TEM.

### 2.5 Animal experiments

A total of 15 female BALB/c mice, aged 6 weeks, were randomly divided into three groups of five mice each. 10 μg VLPs were emulsified with an equal volume of ISA201 adjuvant and then injected intramuscularly with sterile PBS (pH 7.5) as a negative control, and injected with *E. coli*-derived VLPs as a positive control. Blood samples were collected from the lateral canthus of the eye at 14, 28, and 42 days post-immunization (dpi). Mice were euthanized at 42 dpi.

A total of 14 FMDV-negative 2 months-old piglets were procured, and all pigs were housed in a biosafety level 3 (BSL-3) animal facility and randomly divided into three groups, each assigned a separate pig house. The first group of four pigs was administered 2 mL sterile PBS, while the second group of five pigs received 2 mL *E. coli*-derived VLPs (50 μg) as a positive control. The third group was injected with 2 mL yeast-derived VLPs (50 μg). Blood samples were collected at 14 and 28 dpi, respectively. Four weeks after immunization, all pigs were challenged with 2 mL FMDV O strain O/BY/CHA/2010 at 1,000 ID_50_. The animals were observed for 7 days, and the clinical signs were recorded daily.

### 2.6 ELISA for the detection of specific antibodies

Serum samples were analyzed by sandwich ELISA. In brief, rabbit anti-FMDV polyclonal antibody was diluted 1:1000 with 50 mM carbonate buffer (pH 9.6) and coated overnight at 4°C. The next day, the plates were washed three times with PBST and then blocked with 1% BSA at 37°C for 1 h. Subsequently, inactivated O-type FMDV was added to each well and incubated at 37°C for 1 h, washed three times with TBST, after this, added at a 1:100- diluted were added to the plates and incubated at 37°C for 1 h. Then washed three times with PBST and incubated with HRP-conjugated goat anti-pig IgG at 37°C for 1 h. After washed with PBST, the color was developed by adding TMB for 15 min and then terminated with 2 M H_2_SO_4_. The absorbance value was measured at 450 nm.

### 2.7 Neutralizing antibody detection

Neutralizing antibodies in serum samples were determined by microtiter neutralization assay. The serum samples were inactivated at 56°C for 30 min. The serially diluted serum was incubated with the 100 TCID_50_ FMDV O strain O/BY/CHA/2010, incubated at 37°C for 1 h. The mixture was then added to BHK-21 cells in 96-well plates and incubated for 72 h. After incubation, the lesions were observed with an inverted microscope, and the neutralizing antibody titers were calculated using the Reed-Muench method.

### 2.8 Lymphocyte proliferation assay

Mouse spleens were aseptically isolated in a biological safety cabinet and then ground into a single-cell suspension using a 200-mesh nylon mesh. Lymphocyte isolation solution was then added, and the erythrocytes were removed by centrifugation at 500 *g* for 5 min. The cells were then resuspended by adding RPMI1640 culture medium (containing 10% FBS, penicillin/streptomycin) and centrifuged at 500 *g* for 10 min. The cells were then diluted (2 × 106 cells per well). The cells were then added to 96-well plates at a volume of 100 μL. A total of 10 mg/L concentration of Phytohaemagglutinin (PHA) was added as a positive control and untreated lymphocytes as the negative control. RPMI1640 medium was employed as the blank, and 1 μg VLPs-stimulated were used as the experimental group. Four replicates were conducted for each well and incubated at 37°C for 44 h. Subsequently, 10 μL [3-(4,5-dimethylthiazol-2-yl)-5-(3-carboxymethoxyphenyl) –2-(4-sulfophenyl)-2H-tetrazolium, inner salt (MTS)] was added to each well and incubated for 4 h. The absorbance value at 490 nm was measured. The lymphocyte stimulation index (SI) was calculated by dividing the mean value of the test group by that of the negative control group.

### 2.9 Cytokine detection

The IFN-γ levels in serum collected at 28 dpi were quantified using commercially available mouse assay kits (R&D Systems, Minnesota, United States). The procedure was performed according to the manufacturer’s instruction.

### 2.10 Statistics

Data analysis presented in this paper was conducted using GraphPad Prism 8 and statistical significance was performed using one-way analysis of variance. The following symbols are used to indicate statistical significance: “ns” for not significant (*P* = 0.05), “*” for 0.01 = *P* < 0.05, “**” for *P* < 0.01, and “***” for *P* < 0.001.

## 3 Results

### 3.1 Expression of FMDV structural proteins in *P. Pastoris*

**FIGURE 1:**
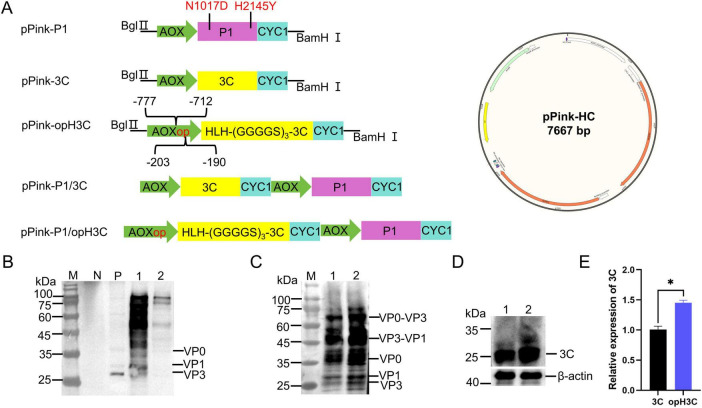
Co-expression of P1 and 3C in *P. Pastoris*. **(A)** Schematic diagram of the plasmid constructs used in this study. pPink-HC plasmid containing the AOX promoter and CYC1 terminator was used to express P1 (pink) and 3C (yellow). **(B)** Western blot (WB) analysis of pPink-P1/3C. Lane N: pPink-HC, Lane P: BHK21-derived VLPs, Lane 1 and 2: pPink-P1/3C. **(C)** WB analysis of pPink-P1/opH3C. Lane M: Marker, Lanes 1 and 2: pPink-P1/opH3C. WB analysis **(D)** and quantification **(E)** of 3C protease in different recombinant strains. “*” for 0.01 = *P* < 0.05.

To express the structural proteins of FMDV in *P. Pastoris*, we constructed plasmids for expressing P1, 3C, and optimized 3C plasmid. We then constructed pPink-P1/3C for co-expression of P1 and 3C. N1017D and H2145Y mutations were introduced into the P1 sequence to enhance the acid stability of VLPs (Caridi et al., 2017). To enhance the catalytic efficiency of 3C, we added the yeast-derived HLH structure, which can anchor to ribosomes, to the N terminal of the 3C gene. By deleting trans-acting sequences and amplifying cis-acting sequences in the AOX promoter, we enhanced its regulation of downstream proteins and constructed the pPink-opH3C plasmid (Figure 1A).

To ascertain if co-expression of P1 and 3C could produce FMDV VLPs in *P. Pastoris*, we screened the pPink-HC, pPink-P1/3C and pPink-P1/opH3C for positive colonies after electrotransformation, followed by induction. After disruption, WB analysis was conducted with anti-FMDV serum as the primary antibody and with BHK-21 cell-derived VLPs as positive controls. No specific bands were detected in the empty plasmid strain. Two specific bands, VP1 (27 kDa) and VP3 (25 kDa), were observed in the positive control, and one specific band, VP0 (35 kDa), was also present. A specific band with a size of 81 KDa was found in pPink-P1/3C, but no clear band was observed at 25-35 kDa (Figure 1B).

Multiple bands were detected in pPink-P1/opH3C. Bands VP3 and VP1 appeared near 25 kDa, a VP0 band near 35 kDa an uncut VP3-VP1 band near 50 kDa and an uncut VP0-VP3 band near 60 kDa (Figure 1C). To determine if P1 cleavage differs between the two strains, we examined 3C expression (Figure 1D). The comparison showed that 3C expression increased about 1.4-fold after optimization (Figure 1E). These results suggest that the 3C in the pPink-P1/3C strain cleaved only a limited quantity of P1 protein. By regulating the 3C protease, 3C expression improved, and cleaved structural proteins (VP0, VP3, and VP1) were detected. Additionally, some cleaved intermediates like VP0–VP1 and VP3–VP1 were present.

### 3.2 Purification and characterization of FMDV VLPs

**FIGURE 2:**
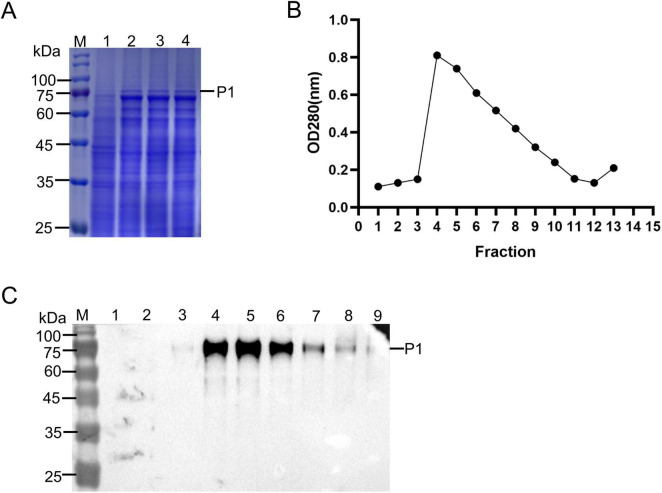
Protein purification of pPink-P1/3C strain. **(A)** SDS-PAGE detection of pPink-P1/3C strain. Lane M: Marker, Lane 1: pPink-HC (negative control), Lane 2–4: pPink-P1/3C induced for 72 h. **(B)** Absorbance detection of proteins after sucrose purification. **(C)** Western blot (WB) analysis of pPink-P1/3C after sucrose purification. The pPink-P1/3C samples were purified by sucrose gradient and divided into 15 fractions from top to bottom. Fractions 1–9 were taken for WB detection.

**FIGURE 3:**
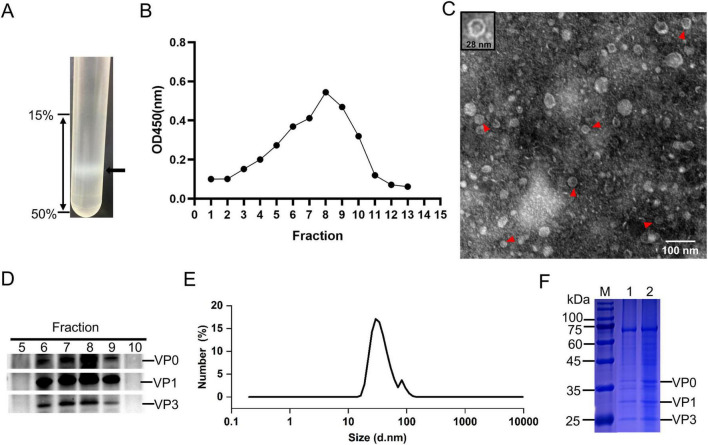
Purification and identification of virus-like particles (VLPs). The pPink-P1/opH3C-induced proteins were purified by sucrose gradient **(A)** and solutions from the top to bottom were divided into 15 fractions and VLPs of each fraction were quantified **(B)** by ELISA. **(C)** Observation of VLPs by transmission electron micros (TEM). **(D)** Western blot (WB) analysis of 5–10 fractions. **(E)** DLS analysis of VLPs. **(F)** SDS-PAGE detection of the purified VLPs.

To determine whether the structure proteins of FMDV expressed in *P. Pastoris*, the cell lysates were collected by centrifugation, and then purified by ultracentrifugation with a 15%–50% sucrose gradient. After 72 h of induction, pPink-P1/3C strain fractions were subjected to SDS-PAGE and compared with the empty vector strain. The results showed P1 was observed above 75 kDa, while the cleaved structure was hard to distinguish (Figure 2A). After sucrose gradient centrifugation, we examined the absorbance at 280 nm of fractions from top to bottom (Figure 2B). The samples in fractions 1–9 were analyzed by WB. Only the uncut precursor P1 was detected, and the cleaved structural proteins were almost undetectable (Figure 2C). Similarly, the VLP fractions were observed in the center of the centrifuge tube after ultracentrifugation with the sucrose gradient (Figure 3A). The samples were then separated from the top to the bottom and the VLPs was quantified by ELISA (Dong et al., 2022). The highest absorbance value was observed in fraction 8, indicating successfully assembled yeast-derived VLPs (Figure 3B). WB analysis showed that VP0, VP3, and VP1 were isolated at the same sucrose density (Figure 3D), suggesting that the VLPs were made of these structural protein subunits. Furthermore, the isolated VLPs were examined using DLS and TEM, which revealed spherical particles with diameters of 28 nm (Figures 3C, E). The VLP samples were subjected to SDS-PAGE, and VP3, VP1, and VP0 were visualized near 25 and 35 kDa (Figure 3F).

### 3.3 Yeast-derived VLPs elicit robust immune responses in mice

**FIGURE 4:**
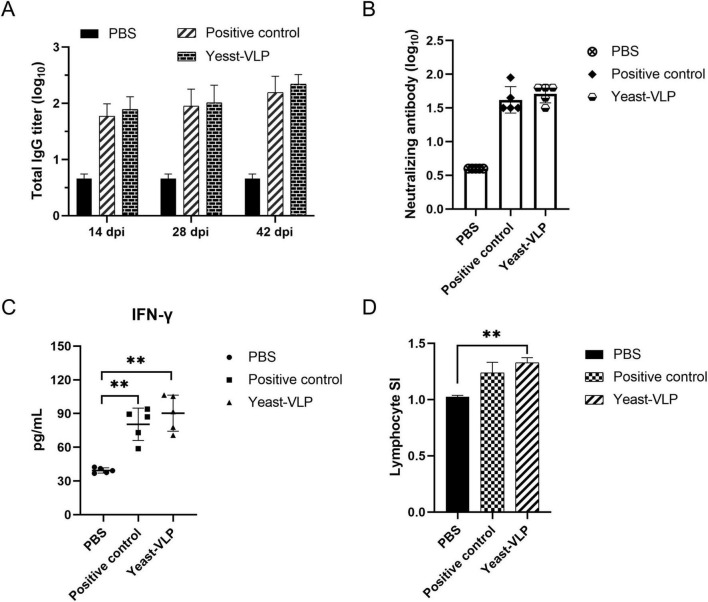
Evaluation of the immune response to virus-like particles (VLPs) in mice. **(A)** Specific antibodies responses by VLPs immunization. **(B)** Neutralizing antibody assay. **(C)** Measurement of IFN-γ levels. Each symbol represents one mouse. **(D)** Lymphocyte proliferation assay. PBS: negative control. Positive control: *E. coli*-derived VLPs. Yeast-VLP: yeast-derived VLPs. “**” for *P* < 0.01.

Purified VLPs were intramuscularly injected into mice to evaluate the immunogenicity of yeast-derived VLPs. Serum samples were collected to measure specific antibody levels. The group injected with VLPs had higher specific antibodies levels than the PBS group. These antibodies were produced by as early as 14 dpi and maintained at 42 dpi (Figure 4A). Neutralizing antibody levels were detected via microplate neutralization assay to determine the capability of VLPs to resist FMDV infection. The results showed that the mice could produce neutralizing antibody levels ranging from 1:32 to 1:64 after 28 dpi (Figure 4B). Moreover, the IFN-γ levels were examined, and the proliferation of splenocytes was assessed after stimulation with VLPs to determine if yeast-derived VLPs can stimulate cellular immune responses in mice. Despite immunization with one-dose VLPs, high levels of IFN-γ were stimulated by the yeast-derived VLPs. Additionally, many lymphocytes were stimulated. Compared with *E. coli*-derived VLPs, the VLPs in this study yielded better results (Figures 4C, D).

### 3.4 Passive protection against FMDV challenge in pigs

**FIGURE 5:**
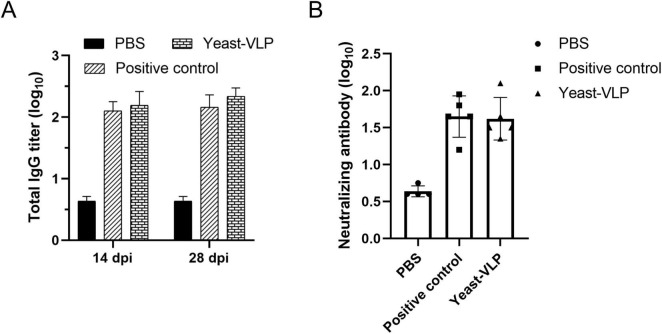
Evaluation of antibody responses by yeast-derived virus-like particles (VLPs) in pigs. Each pig in the immunized group was administered 50 μg VLPs, and serum was collected at 14 and 28 dpi, subsequently diluted fourfold for detection. **(A)** ELISA for FMDV-specific antibodies. **(B)** detection of neutralizing antibodies. Each symbol denotes a single pig. All results are expressed as mean ± standard deviation.

We further evaluated the immunization effect of yeast-derived VLPs on pigs, which are among the natural hosts of FMDV. FMDV-negative pigs were immunized separately with yeast-derived VLPs, *E. coli*-derived VLPs, and PBS. High specific antibodies were detected at 14 dpi; their potency reached 1:256 and persisted up to 28 dpi (Figure 5A). The immunized group exhibited neutralizing antibody levels over 1:22 at 28 dpi (Figure 5B). Fourteen pigs were challenged at 28 dpi, followed by a 7 days observation. By contrast, the PBS group exhibited overt indications of infection within 3 days. One pig in the yeast-derived VLPs group displayed mild clinical signs on its hooves despite a neutralizing antibody titer of 1:22 before the challenge. It was also categorized as unprotected (Table 2). These findings suggest that yeast-derived VLPs can elicit robust humoral and cellular immune responses in pigs and confer over 80% protection against FMDV infection.

**TABLE 2:** The neutralizing titer and protection of pig.

Groups	Yeast-VLP					Positive control					PBS			
No.	1	2	3	4	5	6	7	8	9	10	11	12	13	14
NT	1.35	1.65	1.5	2.1	1.5	1.65	1.95	1.8	1.65	1.2	0.6	0.6	0.75	0.6
Protection	N	Y	Y	Y	Y	Y	Y	Y	Y	Y	N	N	N	N
Percent	80%					100%					0%			

No, number; NT, neutralizing titer.

## 4 Discussion

Foot-and-mouth disease virus is the primary agent responsible for FMD despite decades of efforts to eradicate FMD. Currently, some developed countries in Europe and the United States have eradicated FMDV, but the disease is still a major concern in some developing countries such as Asia, Africa, and America (James and Rushton, 2002; Maradei et al., 2013; Subramaniam et al., 2013). Inactivated vaccines are still the most effective type of vaccine and have played an indelible role in eradicating FMD (Correa Melo et al., 2002; Zhang et al., 2011; Uddowla et al., 2012). However, producing inactivated FMDV vaccines is challenging. It requires costly biocontainment facilities, high-specification laboratories, and manufacturing plants. Also, the virus inactivation process entails a risk of virus leakage or incomplete inactivation (Robinson et al., 2016). These issues is a significant hurdle for economically - weak developing countries.

virus-like particles, self-assembled by viral capsid proteins, can trigger a strong immune response similar to viruses while being extremely safe (Mayr et al., 1999; Cedillo-Barrón et al., 2001; Wang et al., 2016). Available research data indicate that VLP vaccines for FMDV are primarily produced via mammalian cells (Polacek et al., 2013; Mignaqui et al., 2020) or insect cells (Bhat et al., 2013). However, certain technical challenges or limitations are associated with these expression systems.

Despite extensive reports on the production of FMDV VLPs in mammalian and insect cells, detailed yield data remain limited. In contrast, *P. pastoris* offers several advantages for VLP production. It has a short growth cycle, allowing protein harvest within 48 h of induction. Its culture medium is simple, consisting primarily of glycerol, glucose, or methanol, along with inorganic salts. The absence of proteins in the medium simplifies downstream protein purification processes (Karbalaei et al., 2020). Additionally, *P. pastoris* cultivation requires minimal specialized equipment and does not demand the stringent conditions necessary for mammalian or insect cell cultures, which often require expensive serum components and necessitate a strictly sterile environment. Importantly, *P. pastoris* is Generally Recognized as Safe (GRAS), a key attribute for vaccine development and regulatory approval, as demonstrated by the clinical success of human papillomavirus (HPV) and hepatitis B virus (HBV) vaccines produced using this system (McAleer et al., 1984; Sasagawa et al., 1995; Smith et al., 2011). In recent years, FMDV VLPs have been successfully produced using *Saccharomyces cerevisiae* and *Hansenula polymorpha* by co-expressing P1 and 3C. As a low-cost eukaryotic expression system, the *P. Pastoris* has a complete and post-translation modification system and high expression yield. However, only a few studies have produced FMDV VLPs by *P. Pastoris*. The strategy involving the co-expression P1-2A-3C is widely used in eukaryotic expression systems. The strategy utilizes the self-cleavage ability of 2A to generate separate P1 and 3C; cleaves the polyprotein P1 to form VP0, VP3, and VP1 via 3C; and assembles them into VLPs (Kristensen et al., 2018). However, the non-specific cleavage of the 3C protease results in the termination of host transcription (Henikoff, 2008) or cell death (Strong and Belsham, 2004).

This study is the first to produce FMDV VLPs in *P. Pastoris*. P1 and 3C expression cassettes were constructed on the same plasmid and induced within the yeast. Although P1 and 3C could be expressed within the yeast cells, the enzymatic activity of 3C was markedly low. This result is in contrast with the findings for other small RNA viruses, such as EV71 (Zhang et al., 2015) and CA16 (Feng et al., 2016), expressed in *P. Pastoris*. Notably, the 3C protease did not harm *P. Pastoris*, unlike in mammalian and insect cells (Capozzo et al., 2002). Additionally, environment pH has been demonstrated to exert a pronounced influence on the 3C protease activity of EV71 (Cui et al., 2011). Preliminary studies conducted in our laboratory have demonstrated that the 3C protease of FMDV exhibits considerably diminished enzymatic activity at low pH (data not shown). The optimal pH for *P. Pastoris* cell growth is generally acidic (Kim and Kim, 2017), which is bad for the assembly of FMDV VLPs. FMDV is very pH-sensitive (capsid dissociates at pH < 7), while FMDV VLPs are more acid stable than virions (Curry et al., 1995; Stockley et al., 2007). N1017D and H2145Y mutations were introduced to enhance the acid resistance of VLPs. The data demonstrated that this combination of mutations exhibited highly pronounced acid resistance, with the mutated viruses retaining high infectivity at pH up to 5.4 (Caridi et al., 2020). Concurrently, adjustments were made to enhance the cleavage efficacy of the 3C protease. In *P. Pastoris*, the AOX promoter is extremely potent. Some scholars have studied its sequence and identified both cis-acting and trans-acting sequences, which profoundly influence the regulation of AOX promoter (Lin-Cereghino et al., 2006). Consequently, in this study, the cis-acting sequence of 190-203 upstream of AOX was amplified, whereas the trans-acting sequence of 712-777 upstream was deleted, resulting in an approximately 1.4-fold increase in the expression of 3C (Figure 1D). Yeast’s NAC constitutes α and β subunits, with the β subunit being responsible for binding to the ribosome (Beatrix et al., 2000). However, it is the 55 amino acids at the N terminal of the protein that play a pivotal role; they form an HLH structure, with a loop sandwiched between two sides of an α-helix (Wegrzyn et al., 2006). In this study, the HLH structure was added to the N terminus of the 3C to anchor near the exit of the nascent peptide chain of the ribosome increasing its binding to P1 and improving the efficiency of enzyme cutting. Although cleavage was incomplete with intermediate products, VLP assembly wasn’t affected (Figure 1C).

Mice and pigs were immunized with VLPs to verify the antigenicity of yeast-derived VLPs in animals. After immunizing mice, high levels of specific antibodies were detected at 2 weeks, with a much higher neutralizing antibody titer observed at 28 dpi compared with the neutralizing antibody titer observed in the PBS group. Moreover, lymphocyte proliferation assay and cytokine detection demonstrated that the IFN-γ level in mice considerably increased. The stimulation index (SI) of the lymphocytes was elevated by approximately 1.3-fold at 28 dpi, surpassing that of *E. coli*-derived VLPs. This result may be attributed to the fact that yeast is endotoxin-free and causes minimal animal stress. These findings suggest that the yeast-derived VLPs induced Th1- and Th2-type immune responses. Given their relatively simple immune systems and short reproduction cycles, mice are commonly used in preliminary studies of immune mechanism. Research has shown that mice can be used as an evaluation model of FMDV vaccines, and the protection of the vaccine on cattle can be obtained by calculating the specific antibody titer in mice (Gnazzo et al., 2020). As one of the natural hosts of FMDV, the immune effect on pigs was also analyzed. At 28 dpi, the neutralizing antibody titer in pigs was higher than the titer in mice. This might be because pigs are susceptible animals. In susceptible animals, the immune system recognizes the antigen in the vaccine as an external danger signal and rapidly initiates an immune response. B cells specifically recognize the antigen and then differentiate into plasma cells, secreting a large number of neutralizing antibodies. These antibodies can precisely bind to the virus, preventing it from infecting cells, which in turn leads to an increase in the neutralizing antibody titer. Conversely, the immune systems of non-susceptible animals may fail to effectively recognize the vaccine antigen or may mount a weak response upon recognition, resulting in the failure to produce a large number of targeted neutralizing antibodies. High levels of IgG-specific and neutralizing antibodies were stimulated in pigs and showed a positive correlation. After the challenge at 28 dpi, one pig from the VLP-immunized group exhibited clinical symptoms despite having a neutralizing antibody titer of 1:22. Nevertheless, some pigs with a neutralizing antibody level of 1:16 exhibited protection, so the aforementioned pig did not have protection probably because of individual differences.

In conclusion, this study provided a detailed account of the production of FMDV VLPs in *P. Pastoris*, which filled a research gap. The yeast-derived VLPs exhibited a shape and size similar to natural virions, induced high levels of antibodies, and provided protection against FMDV. This work offers new insights for the development of FMDV VLP vaccine and the expression of exogenous proteins by *P. Pastoris.*

## Data Availability

The datasets presented in this study can be found in online repositories. The names of the repository/repositories and accession number(s) can be found in the article/Supplementary material.
