# Supplementary material for: Evaluation of the immune effect of foot-and-mouth disease virus-like particles derived from Pichia Pastoris on mice and pigs
Source: Front Microbiol. 2025 Apr 14;16:1551395. doi: 10.3389/fmicb.2025.1551395 (PMC12034696; doi:10.3389/fmicb.2025.1551395)
Supplement: Supplementary file 1 [file Data_Sheet_1.docx]

**Evaluation of the immune effect of foot-and-mouth disease virus-like particles derived from *Pichia Pastoris* on mice and pigs**

**Zhiyao Li^1,2^, Manyuan Bai^2^, Shuanghui Yin^2^, Yan Yang^3^, Hu Dong^2^, Zhidong Teng^2^, Shiqi Sun^2,4^, Endong Bao^1*^, Huichen Guo^2,4*^**

^1^College of Veterinary Medicine, Nanjing Agricultural University, Nanjing 210095, China.

^2^State Key Laboratory for Animal Disease Control and Prevention, College of Veterinary Medicine, Lanzhou University, Lanzhou Veterinary Research Institute, Chinese Academy of Agricultural Sciences, Lanzhou 730000, China.

^3^Agriculture and Rural Bureau of Yugur Autonomous County of Sunan, Zhangye 734400, China.

^4^Gansu Province Research Center for Basic Disciplines of Pathogen Biology, Lanzhou 730046, China.

*** Correspondence**: Endong bao, Nanjing Agricultural University, Nanjing 210095, China; E-mail: b_endong@njau.edu.cn.

*** Correspondence:** Huichen Guo, Lanzhou Veterinary Research Institute, Chinese Academy of Agricultural Sciences, Lanzhou 730000, China; E-mail: guohuichen@caas.cn.

Optimized P1 gene sequence:2202 bp

GGTGCTGGTCAATCTTCTCCAGCTACTGGTTCTCAAAACCAATCTGGTAATACTGGTTCTATTATTAACAACTACTACATGCAACAATACCAAAACTCTATGGATACTCAATTGGGAGATAACGCTATTTCTGGTGGTTCTAATGAAGGTTCTACTGATACTACTTCTACTCATACTACTAACACTCAAAACAACGATTGGTTCTCTAAGTTGGCTTCTTCTGCTTTTTCTGGTTTGTTCGGTGCTTTGTTGGCTGATAAGAAAACTGAAGAGACTACTTTGTTGGAAGATAGAATTTTGACTACTAGAAACGGTCATACTACTTCTACTACTCAATCTTCTGTTGGTATTACTCACGGTTACGCTACTGCTGAGGATTTCGTTAACGGTCCAAATACTTCTGGTTTGGAAACTAGAGTTATTCAAGCTGAGAGATTTTTCAAGACTCATTTGTTCGATTGGGTTACTTCTGATCCATTCGGTAGATACTATTTGTTGGAATTGCCTACTGATCACAAAGGTGTTTATGGTTCTTTGACTGATTCTTACGCTTATATGAGAAATGGTTGGGATGTTGAAGTTACTGCTGTTGGTAACCAATTCAACGGTGGTTGTTTGTTGGTTGCTATGGTTCCTGAGTTGTGTTCTATTGAACAAAGAGAGTTGTTCCAATTGACTTTGTTCCCATATCAATTCATTAACCCTAGAACTAACATGACTGCTCACATTAAAGTTCCATTTGTTGGTGTTAACAGATACGATCAATACAAGGTTCATAAACCTTGGACTTTGGTTGTTATGGTTGTTGCTCCATTGACTGTTAATACTGAAGGTGCTCCTCAAATTAAGGTTTATGCTAACATTGCTCCAACTAATGTTCACGTTGCTGGTGAATTTCCATCTAAAGAGGGTATTTTCCCTGTTGCTTGTTCTGATGGTTACGGTGGTTTGGTTACTACTGATCCAAAGACTGCTGATCCTGTTTATGGTAAAGTTTTTAACCCACCTAGAAACATGTTGCCTGGTAGATTCACTAACTTGTTGGATGTTGCTGAGGCTTGTCCAACTTTCTTGCATTTCGATGGAGATGTTCCTTACGTTACTACTAAGACTGATTCTGATAGAGTTTTGGCTCAATTTGATTTGTCTTTGGCTGCTAAACATATGTCTAACACTTTCTTGGCTGGTTTGGCTCAATACTACACTCAATACTCTGGTACTGTTAATTTGCACTTCATGTTCACTGGTCCAACTGATGCTAAGGCTAGATACATGATTGCTTATGCTCCACCTGGTATGGAACCACCTAAAACTCCTGAGGCTGCTGCTCATTGTATTCACGCTGAATGGGATACTGGTTTGAACTCTAAGTTTACTTTCTCTATTCCATACTTGTCTGCTGCTGATTACGCTTATACTGCTTCTGATGCTGCTGAGACTACTAATGTTCAAGGTTGGGTTTGTTTGTTTCAAATTACTCACGGTAAAGCTGAAGGAGATGCTTTGGTTGTTTTGGCTTCTGCTGGTAAAGATTTCGAGTTGAGATTGCCAGTTGATGCTAGACAACAAACTACTTCTACTGGTGAATCTGCTGATCCTGTTACTACTACTGTTGAAGACTATGGTGGTGAGACTCAAGTTCAAAGAAGACATCACACTGATGTTTCTTTTATTTTGGATAGATTCGTTAAGGTTACTCCAAAGGATTCTATTAATGTTTTGGATTTGATGCAAACTCCTTCTCATACTTTGGTTGGTGCTTTGTTGAGAACTGCTACTTACTATTTTGCTGATTTGGAGGTTGCTGTTAAGCACAAAGGAGATTTGACTTGGGTTCCAAACGGTGCTCCTGTTGCTGCTTTGGATAACACTACTAATCCAACTGCTTACCATAAGGCTCCTTTGACTAGATTGGCTTTGCCATATACTGCTCCTCACAGAGTTTTGGCTACTGTTTACAACGGTGAGTGTAAGTATGCTGAGGGTTCTTTGCCAAATGTTAGAGGAGATTTGCAAGTTTTGGCTCAAAAAGCTGCTAGACCATTGCCTACTTCTTTCAATTACGGTGCTATTAAGGCTACTAGAGTTATTGAATTGTTGTACAGAATGAAAAGAGCTGAGACTTATTGTCCAAGACCTTTGTTGGCTGTTCATCCATCTGCTGCTAGACACAAGCAAAAGATTGTTGCTCCTGTTAAACAA

Optimized 3C gene sequence:639 bp

TCTGGTGCTCCACCTACTGATTTGCAAAAGATGGTTATGGGTAACACTAAACCAGTTGAGTTGATTTTGGATGGTAAAACTGTTGCTATTTGTTGTGCTACTGGTGTTTTTGGTACTGCTTACTTGGTTCCTAGACATTTGTTCGCTGAAAAGTACGATAAGATCATGTTGGATGGTAGAACTATGACTGATTCTGATTACAGAGTTTTCGAATTTGAGATTAAGGTTAAGGGTCAAGATATGTTGTCTGATGCTGCTTTGATGGTTTTGCATCGTGGTAACAGAGTTAGAGATATCACTAAGCACTTCAGAGATACTGCTAGAATGAAGAAAGGTACTCCAGTTGTTGGTGTTATTAACAATGCTGATGTTGGTAGATTGATTTTCTCTGGTGAAGCTTTGACTTACAAGGATATCGTTGTTTGTATGGATGGAGATACTATGCCTGGTTTGTTCGCTTACAAGGCTGCTACTAAAGCTGGTTATTGTGGTGGTGCTGTTTTGGCTAAAGATGGTGCTGATACTTTTATTGTTGGTACTCATTCTGCTGGTGGTAACGGTGTTGGTTATTGTTCTTGTGTTTCTAGATCCATGTTGCAAAAGATGAAAGCTCACATTGATCCAGAGCCTCATCACGAA

Optimized HLH gene sequence: 165 bp

ATGCCAATTGACCAAGAAAAATTAGCTAAGCTACAAAAGTTGTCTGCTAACAACAAAGTTGGTGGTACTAGAAGAAAGCTTAACAAGAAGGCAGGCTCTTCTGCCGGTGCCAACAAGGATGACACCAAGTTGCAAAGTCAATTAGCTAAGTTGCACGCTGTCACC
